# Responses of Linear and Cyclic Electron Flow to Nitrogen Stress in an N-Sensitive Species *Panax notoginseng*

**DOI:** 10.3389/fpls.2022.796931

**Published:** 2022-02-15

**Authors:** Zhu Cun, Hong-Min Wu, Jin-Yan Zhang, Sheng-Pu Shuang, Jie Hong, Jun-Wen Chen

**Affiliations:** ^1^College of Agronomy and Biotechnology, Yunnan Agricultural University, Kunming, China; ^2^Key Laboratory of Medicinal Plant Biology of Yunnan Province, Yunnan Agricultural University, Kunming, China; ^3^National & Local Joint Engineering Research Center on Germplasm Innovation & Utilization of Chinese Medicinal Materials in Southwestern China, Yunnan Agricultural University, Kunming, China

**Keywords:** nitrogen, photoinhibition, photoprotection, electron transport, *Panax notoginseng*

## Abstract

Nitrogen (N) is a primary factor limiting leaf photosynthesis. However, the mechanism of N-stress-driven photoinhibition of the photosystem I (PSI) and photosystem II (PSII) is still unclear in the N-sensitive species such as *Panax notoginseng*, and thus the role of electron transport in PSII and PSI photoinhibition needs to be further understood. We comparatively analyzed photosystem activity, photosynthetic rate, excitation energy distribution, electron transport, OJIP kinetic curve, P700 dark reduction, and antioxidant enzyme activities in low N (LN), moderate N (MN), and high N (HN) leaves treated with linear electron flow (LEF) inhibitor [3-(3,4-dichlorophenyl)-1,1-dimethyl urea (DCMU)] and cyclic electron flow (CEF) inhibitor (methyl viologen, MV). The results showed that the increased application of N fertilizer significantly enhance leaf N contents and specific leaf N (SLN). Net photosynthetic rate (*P*_n_) was lower in HN and LN plants than in MN ones. Maximum photochemistry efficiency of PSII (*F*_v_/*F*_m_), maximum photo-oxidation P700^+^ (*P*_m_), electron transport rate of PSI (ETRI), electron transport rate of PSII (ETRII), and plastoquinone (PQ) pool size were lower in the LN plants. More importantly, K phase and CEF were higher in the LN plants. Additionally, there was not a significant difference in the activity of antioxidant enzyme between the MV- and H_2_O-treated plants. The results obtained suggest that the lower LEF leads to the hindrance of the formation of ΔpH and ATP in LN plants, thereby damaging the donor side of the PSII oxygen-evolving complex (OEC). The over-reduction of PSI acceptor side is the main cause of PSI photoinhibition under LN condition. Higher CEF and antioxidant enzyme activity not only protected PSI from photodamage but also slowed down the damage rate of PSII in *P. notoginseng* grown under LN.

## Introduction

Photosynthesis is one of the most important physiological and biochemical reactions in nature (Berry et al., 2013; Martin et al., 2018). Nitrogen (N) is regarded as a necessary component of numerous biomolecules, such as Rubisco (Ribulose-1,5-bisphosphate carboxylase/oxygenase), chlorophyll (Chl), and photosynthetic proteins (Evans and Clarke, 2019). Photosynthetic capacity is closely related to leaf N content (Makino and Osmond, 1991). The N fertilizer application might increase N content, light harvesting capacity, and *P*_n_ (net photosynthetic rate) of leaves (Evans and Clarke, 2019). This has been confirmed by the performance observed in *Oryza sativa* and *Arabidopsis thaliana* grown under high N (HN) application (Perchlik and Tegeder, 2018; Hou et al., 2019). However, plants exposed to long-term N deficiency would suffer an imbalance between the capability for absorbing light energy and consuming this excess light energy, resulting in the generation of reactive oxygen species (ROS) (Yamori et al., 2016; Che et al., 2020; Mu and Chen, 2021). Photosystem II (PSII) is susceptible to HN and low N (LN) (Cisse et al., 2020; Zhang Z. et al., 2021). HN and LN can inhibit either on the acceptor side or on the donor side of PSII, resulting in damaging PSII functions. The donor side of PSII in *Porphyridium cruentum*, *O. sativa*, and *Vitis labrusca* were seriously impacted by N deficiency, leading to the inactivation of the oxygen-evolving complex (OEC) and the reduced photochemical efficiency (Chen and Cheng, 2003; Zhao et al., 2017; Tantray et al., 2020). HN supply increases the PSII photoinhibition in *O. sativa* and *Chenopodium quinoa* because it does not sustain the balance of light absorption and utilization and consequently accumulates large amounts of H_2_O_2_ at PSII (Bascuñán-Godoy et al., 2018; Cisse et al., 2020). Diverse environmental stress can also result in the impairment of photosystem I (PSI) (Ivanov et al., 2015; Lima-Melo et al., 2019). The activity of PSI can be inhibited in *Nicotiana tabacum* grown under N stress (Yue et al., 2021). However, the mechanism of inhibition of PSII and PSI by N stress is not well-understood.

Plants are susceptible to photodamage under N stress; however, they can dissipate excessive energy through non-photochemical quenching (NPQ) pathway, the xanthophyll cycle, and state transitions (Takahashi and Badger, 2011; Pinnola and Bassi, 2018). Photoprotection of diatoms are elevated through NPQ during N starvation (Liefer et al., 2018; Li Z. et al., 2021). Photosynthetic electron transport [linear electron flow (LEF) and cyclic electron flow (CEF)] in the thylakoid membrane is very important for photoprotection in plants grown under N stress (Shikanai, 2007; Yamamoto et al., 2021). CEF and LEF are essential for balancing the production ratio of ATP/NADPH and for protecting photosystems from impairment by over-reduction of chloroplast stroma (Lu et al., 2020). PSII photodamage is avoided in *Phaseolus vulgaris* and *Camellia sinensis* grown under N limitation through enhancing CEF (Antal et al., 2010; Lin et al., 2016). Plants also effectively dissipated over-excitation by the transition of LEF to CEF, which relieves the excitation pressure in photosystems and diminishes ROS generation (Tu et al., 2016; Podgórska et al., 2020). This was confirmed in *Solanum lycopersicum* grown under high temperature and high light treated with LEF inhibitor: 3-(3,4-dichlorophenyl)-1,1-dimethyl urea (DCMU) and CEF inhibitor: methyl viologen (MV) (Lu et al., 2017). CEF (CEF-PSI) and moderate photoinhibition of PSII are two main protecting mechanisms of PSI photoinhibition (Zhang et al., 2014; Huang et al., 2016a). CEF-PSI alleviates the over-reduction of acceptor side of PSI and the generation of superoxide anion, and also protects PSI from photoinhibition (Huang et al., 2012a). This has also been confirmed by the results observed in *P. vulgaris* and *C. sinensis* grown under N stress (Antal et al., 2010; Lin et al., 2016). On the other hand, the excess electron flow from PSII causes photoinhibition of PSI (Scheller and Haldrup, 2005; Rochaix, 2011). When DCMU is used to block the electron transport from PSII to PSI, the photoinhibition of PSI would not be observed in chilled *Solanum tuberosum, Cucumis sativus*, and *Spinacia oleracea* (Sonoike, 1996). Photoinhibition of *A. thaliana pgr5*-mutant and *Psychotria rubra* by high light can be alleviated when the addition of DCMU restricts the electron flow from PSII to PSI (Suorsa et al., 2012; Huang et al., 2016b). PSI photoinhibition is mainly dependent on the electron flow from PSII to PSI. Thus, photosynthetic electron transport plays an important role in photoprotection in the N-stress plants.

*Panax notoginseng* (Burkill) F. H. Chen (Sanqi in Chinese) is a typically shade-tolerant and N-sensitive species from the family of Araliaceae (Yang et al., 2008; Chen J.W. et al., 2016; Ou et al., 2020; Zhang Q.H. et al., 2020; Zhang J.Y. et al., 2021). Excessive application of N fertilizer is a problem in *P. notoginseng* production (Ou et al., 2020). N application rate in conventional cultivation of *P. notoginseng* is 450 kg ha^–1^ (Xia et al., 2016), which not only exceeded its demand (Ou et al., 2011) but also increased root decay and mortality rate; thus, *P. notoginseng* has been commonly believed to be the N-sensitive species (Zheng et al., 2017; Wei et al., 2018; Zhang J.Y. et al., 2020). There is an elevation in leaf biomass and Chl content of *P. notoginseng* accompanying with the decrease of *P*_n_ under HN application (Li, 2017; Cun et al., 2020). In our previous work, it has been recorded that the photosynthetic performance was significantly suppressed in HN- and LN-grown *P. notoginseng* (Cun et al., 2020; Zhang J.Y. et al., 2020; Cun et al., 2021). *P. notoginseng* grown under LN condition reduces the photochemical efficiency of PSII through NPQ, xanthophyll cycle, antioxidant pathways, Chl degradation, and nitrate metabolism (Zhang J.Y. et al., 2020). Photosynthetic capacity is reduced in *P. notoginseng* grown under HN application mainly due to the inactivation of Rubisco (Cun et al., 2021). In addition, the activation of CEF cannot completely protect PSII donor side from damage in *P. notoginseng* under HN, but can attenuate PSI photodamage (Cun et al., 2021). However, the mechanism of the PSI and PSII photoinhibition in the N-sensitive species *P. notoginseng* under N stress is still not completely understood, and the role of electron transport of PSII and PSI in photoinhibition needs to be further investigated.

In the present study, the photosystem activity, photosynthetic rate, excitation energy distribution, electron transport, OJIP kinetic curve, antioxidant enzyme activity, and P700 dark reduction curves were examined in the LN, MN, and HN plants in the presence of DCMU and MV. The spraying of DCMU and MV would inhibit LEF and CEF, respectively. We hypothesized that (i) LEF-mediated damage to the PSII OEC is the main cause of PSII photoinhibition under LN condition; (ii) over-reduction of PSI acceptor side is the main cause of PSI photoinhibition under LN; and (iii) the activation of CEF might protect PSI from photodamage under LN.

## Materials and Methods

### Plant Materials and Growth Conditions

The experiment site was situated in the teaching and experimental farm of Yunnan Agricultural University in Kunming, China (102°45′E, 25°08′N). The physical and chemical properties of the raw soils used in the present study were as follows. pH 6.84, organic matter 3.18%, total N 0.17%, total phosphorus 0.23%, total potassium 0.24%, available potassium 127.32 mg g^–1^, and available phosphorus 11.04 mg kg^–1^. In January 2019, healthy 1-year-old *P. notoginseng* seedlings (purchased from the Wenshan Miao Xiang *P. notoginseng* Industrial Co., Ltd., Wenshan, China) were sterilized by 50% chloroisobromine cyanuric acid 1,000-fold dilution for 10 min and transplanted in pots (32 cm × 19 cm × 21 cm) with each containing three rootstocks, 140 pots per treatment were arranged. Five independent experiments were conducted in an environmentally controlled house with irradiance of about 10% full sunlight. Based on the application of 225 kg of phosphorus (P_2_O_5_) and 225 kg of potassium (K_2_O) per hectare in *P. notoginseng* production, three nitrogen levels, LN (without N addition), moderate nitrogen (MN, 225 kg ha^–1^ N), and HN(450 kg ha^–1^ N), were designed for the present study. Fertilizer was applied in four times a year, and N was supplied in April, May, July, and August, respectively, each year. In August 2020, photosynthetic parameters were measured in mature leaves grown under different N regimes.

### Steady-State Photosynthetic Gas Exchange Rate

The steady-state gas exchange parameters were measured by the photosynthesis system (Li-6400, Li-COR, Lincoln, NE, United States). The blue light ratio of the instrument, leaf temperature, and CO_2_ in the chamber were maintained at 10%, 25°C, and 400 μmol mol^–1^, respectively. Leaves were adapted under light (500 μmol⋅photons⋅m^–2^ s^–1^) for 10 min before measurement, and the automatic measurement was started after the data had stabilized. Subsequently, the steady-state photosynthetic gas exchange rate was recorded after exposure for 2–3 min to each light intensity (500, 300, 200, 150, 100, 80, 70, 60, 50, 40, 30, and 20 μmol⋅photons⋅m^–2^ s^–1^; five replicates per treatment). According to the study of Webb et al. (1974), the relationship between *P*_n_ and photosynthetic photon flux density (PPFD) was fitted (*n* = 5), *P*_n_ = *P*_max_ − *P*_max_C_0_e^–α^ ^PPFD/^*^P^*^max^ (Bassman and Zwier, 1991).

### Photosystem I and Photosystem II Measurements

Biophysical parameters were measured simultaneously by Dual PAM 100 (Heinz Walz GmbH, Effeltrich, Germany). To measure the light response of the steady-state PSI and PSII parameters at various light intensities, healthy matured leaves were illuminated at a saturating light of 172 μmol⋅photons⋅m^–2^ s^–1^ for 20 min. Subsequently, photosynthetic capacity was evaluated at 30 s intervals at PPFD of 501, 330, 214, 172, 94, 36, 18, 10, and 0 μmol⋅photons⋅m^–2^ s^–1^. The maximum quantum yield of PSII, *F*_v_/*F*_m_ = (*F*_m_ − *F*_o_)/*F*_m_, was measured to monitor PSII activity. *F*_o_ and *F*_m_ are the minimum and maximum fluorescence measured after 2 h dark adaptation. On the other hand, the PSI photosynthetic parameters were evaluated by Dual PAM 100 based on P700 oxidation signal (Klughammer and Schreiber, 2008). The maximum photo-oxidation P700^+^ (*P*_m_) was determined using a saturation pulse of far-red pre-irradiation light; the determination of *P*_m_‘ differs from *P*_m_, in that *P*_m_ was determined using actinic light rather than far-red light (Takagi et al., 2017). Additionally, treated leaves were illuminated at a saturating light of 172 μmol⋅photons⋅m^–2^ s^–1^ for 20 min to measure the light energy allocation and electron transport parameters. The formulae of Chl fluorescence parameters were shown in Supplementary Table 1 (Krall and Edwards, 1992; Strasser and Srivastava, 1995; Yamori et al., 2011; Huang et al., 2012b) (*n* = 5).

### Chl *a* Fluorescence Measurement and OJIP Transient Analyses

The Chl *a* fluorescence was measured simultaneously by Dual PAM 100. The plants were kept in the dark for 2 h to achieve a dark-adapted state with open reaction centers (RCs). When leaf was illuminated by a high density of actinic light (10,000 μmol⋅photons⋅m^–2^ s^–1^), the fast fluorescence kinetics was recorded from 10 μs to 1 s. *F*_O_, *F*_J_, *F*_I_, and *F*_P_ correspond to the relative fluorescence intensity at the time points of 20 μs, 2, 30, and 300 ms, respectively. The point of time corresponding to 300 μs on the OJIP kinetic curves was defined as the K characteristic points. The ratio of variable *F*_K_ to the amplitude (*F*_J_ – *F*_O_) was calculated as: *W*_K_ = (*F*_K_ − *F*_O_)/(*F*_J_ − *F*_O_) (Li P. et al., 2009). The parameter *W*_K_ is an indicator of damage to the OEC activity (Strasser, 1997; Strasser et al., 2000, 2004; De Ronde et al., 2004; Li P. et al., 2009). The parameter *PI*_ABS_ is a performance index (potential) for energy conservation from excitation to the reduction of intersystem electron acceptors (Strasser et al., 2000, 2004). For a detailed analysis of the whole fluorescence kinetics, different normalizations and kinetic differences in calculations were undertaken. Abbreviations, formulas, and definitions of the JIP-test parameters used in the present study were presented in Supplementary Table 1 (Strasser et al., 2000; Kumar et al., 2020; Devadasu et al., 2021) (*n* = 5).

### Measurement of Plastoquinone Pools

The P700 signal was determined during single-turn saturation pulse [ST, 50 ms, plastoquinone (PQ) pools being oxidized] followed by multiple-turn saturation pulse (MT, 50 ms, PQ pools fully reduced) in the presence of far-red background light (Savitch et al., 2001; Luo et al., 2021). The complementary area between the P700 oxidation curve after a single-turnover and multiple-turnover excitations and the stationary level of P700^+^ under far-red light represents the single-turn and multiple-turn areas, respectively. These were used to calculate the functional pool sizes of the intersystem electrons relative to P700 as: e^–/P700^ = multiple-turn areas/single-turn areas (Savitch et al., 2001) (*n* = 5).

### Photoinhibitory Treatments

To determine the roles of electron transport from PSII and PSI redox state in PSI photoinhibition in *P. notoginseng* grown under different N levels, leaves were sprayed with H_2_O, MV and DCMU in darkness. The electron transport inhibitor concentration with the greatest inhibition of PSI and PSII activity was selected for the treatment of *P. notoginseng* leaves (Supplementary Figure 1). In August 2020, 3-year-old *P. notoginseng* leaves were sprayed with H_2_O, DCMU (180 μm), and MV (150 μm) for 12 h in darkness. The treated leaves were allowed to be air-dried for 10 min to remove excess water, and then steady-state photosynthetic gas exchange rate, Chl fluorescence, and PQ pools were measured (*n* = 5).

### Determination of Biomass and N Content

After completing photosynthetic measurement, the tagged plants were measured for biomass (they are the dry weight of root, stem, and leaves). The N content was determined by the Kjeldahl method (Stafilov et al., 2020). Specific leaf N (SLN, g m^–2^) was calculated (*n* = 5). Based on the biomass and N content, the N use efficient (NUE) was calculated as: NUE (kg kg^–1^) = yield (underground dry weight)/plant N accumulation (Ning et al., 2012; Wu et al., 2016) (*n* = 5).

### Photosynthetic Pigment Analyses

About 0.5 g fresh leaves extracted by 25 ml extraction mixture [acetone and anhydrous alcohol were mixed (v/v 1:1)] were placed under dark condition for 24 h. Absorbance were performed at wavelengths of 663 and 645 nm, respectively. Then pigment contents were calculated according to the method of Pérez-Patricio et al. (2018) (*n* = 5).

### Leaf Rubisco Activity

Rubisco activity was evaluated according to Parry et al. (1997). The extraction solution was prepared as follows: 50 mM Tris–HCl (pH 8.0), 10 mM β-mercaptoethanol, 12.5% glycerol (v/v), 1 mM ethylenediaminetetraacetic acid (EDTA)–Na_2_, 10 mM MgCl_2_ and 1% polyvinylpyrrolidone (PVP)-40 (m/v). Extracts were clarified by centrifugation (8,000 × *g* at 4°C for 10 min), and the supernatant was immediately assayed for Rubisco activity (*n* = 5).

### Determination of Antioxidant Enzyme Activity

Determination of antioxidant enzyme activity was measured as described in Fatemi et al. (2021). After photoinhibitory treatments, the leaves were harvested and immediately frozen in liquid N, and stored at –80°C. Leaves (ca. 0.1 g) were crushed into a fine powder using a mortar and pestle under liquid N. Cell-free homogenates for antioxidant enzyme assays were prepared. Superoxide dismutase (SOD) activity was measured by the nitroblue tetrazolium (NBT) reduction method (Nebot et al., 1993). Glutathione peroxidase (POD) activity was measured by the guaiacol oxidation method (Polle et al., 1994; Shi et al., 2010). Catalase (CAT) activity was measured by the UV absorption method (Chen et al., 2011).

### Statistical Analysis

All data obtained were analyzed with one-way ANOVA with statistical software (IBM SPSS 20.0, IBM Corp., Armonk, NY, United States). Means were separated by the Duncan’s test, and *p-*values less than 0.05 were considered statistically significant, where the data were tested to confirm normality and the variables were present as the mean ± SD (*n* = 5). Subsequently, SigmaPlot 12.5 (Systat Software Inc., San Jose, CA, United States) and GraphPad Prism 8.0 (CA, United States) software were used to plot.

## Results

### Effect of N Regimes on Biomass Partitioning, N, and Chl Content

**FIGURE 1:**
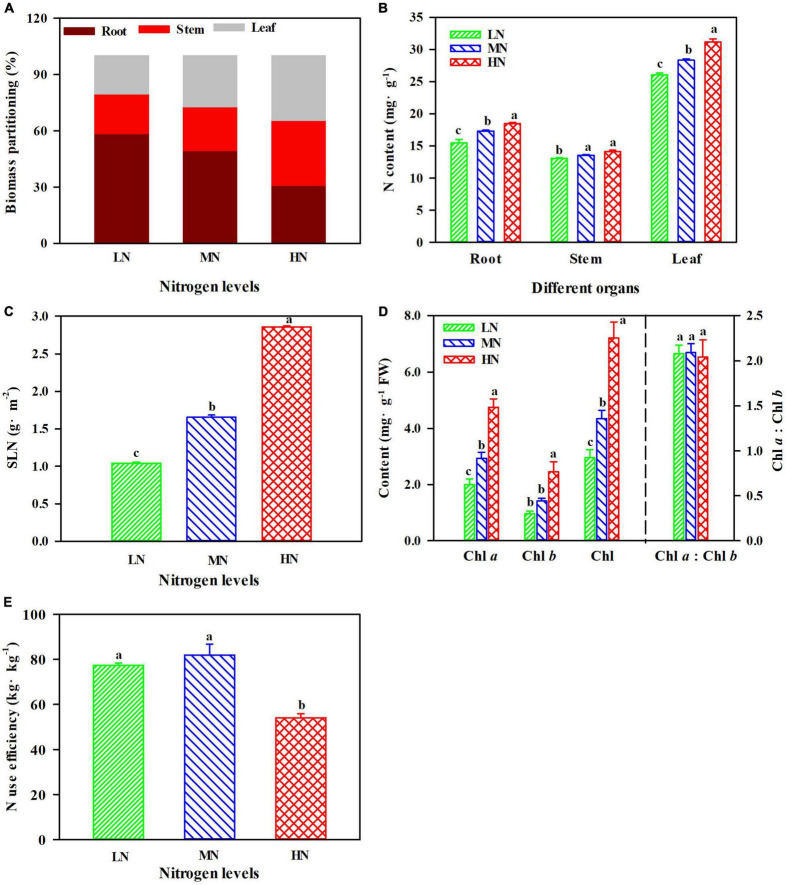
The effect of nitrogen (N) regimes on biomass partitioning **(A)**, N content **(B)**, specific leaf N (SLN) **(C)**, chlorophyll content **(D)**, and N use efficiency **(E)** in *Panax notoginseng*. Values for each point were mean ± SD (*n* = 5). Different letters indicate significant differences among treatments (ANOVA; *p* < 0.05).

There were significant differences in biomass allocation under N regimes. Leaf and stem biomass were greater when plants were exposed to HN compared with MN and LN, but the maximum value of root biomass was obtained in the LN plants (Figure 1A). N contents of different organs were significantly increased with the increase of N levels (Figure 1B), and the N content in leaves was significantly higher than that in roots and stems (Figure 1B; *p* < 0.05). Additionally, SLN increased by 72.34% in HN plants compared with MN plants, and SLN declined by 40.00% in the LN plants (Figure 1C; *P* < 0.05). The contents of Chl *a*, Chl *b*, and Chl were significantly increased with the increase of N levels, but the ratio of Chl *a* to Chl *b* (Chl *a*: Chl *b*) was not significantly different among three treatments (Figure 1D). N use efficiency (NUE) did not show apparent differences between LN and MN plants, but the minimum values of NUE were generally recorded in the HN plants (Figure 1E; *p* < 0.05).

### Effect of N Regimes on Photosynthetic Rate, Photosystem, and Rubisco Activity

**FIGURE 2:**
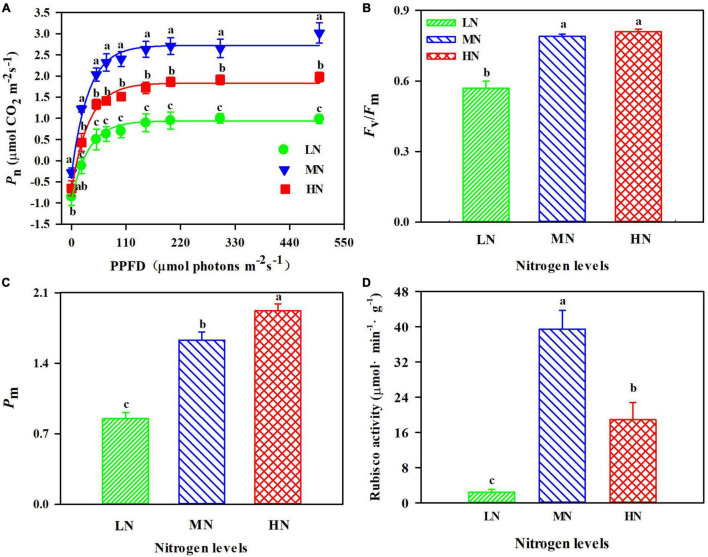
**(A)** Response of net photosynthetic rate (*P*_n_) to photosynthetic photon flux density (PPFD) in *Panax notoginseng* grown under low nitrogen (LN, 0 kg hm^– 2^), moderate nitrogen (MN, 225 kg hm^– 2^), and high nitrogen (HN, 450 kg hm^– 2^). The effects of nitrogen regimes on PSI **(B)**, PSII **(C)**, and Rubisco activity **(D)** of *P. notoginseng*. *P*_m_ is the maximum photo-oxidation P700^+^; *F*_v_/*F*_m_ is the maximum efficiency of PSII photochemistry. Values for each point were mean ± SD (*n* = 5). Different letters among nitrogen regimes indicate significant difference (*p* < 0.05).

The leaf exhibited a significant difference in response of *P*_n_ to incident PPFD within N regimes (Figure 2A). *P*_n_ were highest in MN-grown plants (Figure 2A). LN-grown leaves were dramatically reduced in activity of PSI (*P*_m_, maximum photo-oxidation P700^+^) and PSII (*F*_v_/*F*_m_, maximum photochemistry efficiency of PSII) (Figures 2B,C). *F*_v_/*F*_m_ did not show apparent difference between MN and HN plants (Figure 2B). Moreover, MN treatment exhibited 52.11–93.84% more Rubisco activity than others (Figure 2D; *p* < 0.05).

### Effects of 3-(3,4-Dichlorophenyl)-1,1-Dimethyl Urea and Methyl Viologen on Photoinhibition of Photosystem I and Photosystem II Under N Regimes

**FIGURE 3:**
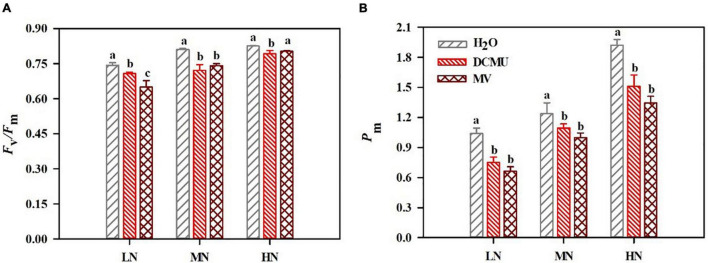
Effects of 3-(3,4-dichlorophenyl)-1,1-dimethyl urea (DCMU) and methyl viologen (MV) on *F*_v_/*F*_m_ **(A)** and *P*_m_ **(B)** in leaves of *Panax notoginseng* grown under different nitrogen regimes. After infiltration with chemical reagents (H_2_O, DCMU, and MV) in darkness for 12 h. *F*_v_/*F*_m_, and *P*_m_ were measured as described in the section “Materials and Methods.” Values for each point were mean ± SD (*n* = 5). Different letters indicate significant differences among treatments (*p* < 0.05, one-way ANOVA).

The *F*_v_/*F*_m_ and *P*_m_ decreased in samples were treated with DCMU and MV (Figure 3). Compared with the H_2_O-treated plants, *F*_v_/*F*_m_ decreased by 4.8 and 12.5% in the DCMU- and MV-treated LN-grown plants (Figure 3A). In LN-grown plants treated with DCMU and MV, the value of *P*_m_ reduced by 27.9 and 36.3%, respectively (Figure 3B). In MN-grown plants treated with DCMU and MV, the value of *F*_v_/*F*_m_ reduced by 11.0 and 8.5%, respectively (Figure 3A). *P*_m_ decreased by 11.6 and 19.3% in the DCMU- and MV-treated MN-grown plants (Figure 3B). *F*_v_/*F*_m_ declined by 4.0 and 2.8% in HN-grown plants treaded with DCMU and MV (Figure 3A). Meanwhile, *P*_m_ reduced by 21.4 and 30.1% in the DCMU- and MV-treated HN-grown plants (Figure 3B).

### 3-(3,4-Dichlorophenyl)-1,1-Dimethyl Urea and Methyl Viologen Induce Changes in Photosynthetic Electron Transport Under N Regimes

**FIGURE 4:**
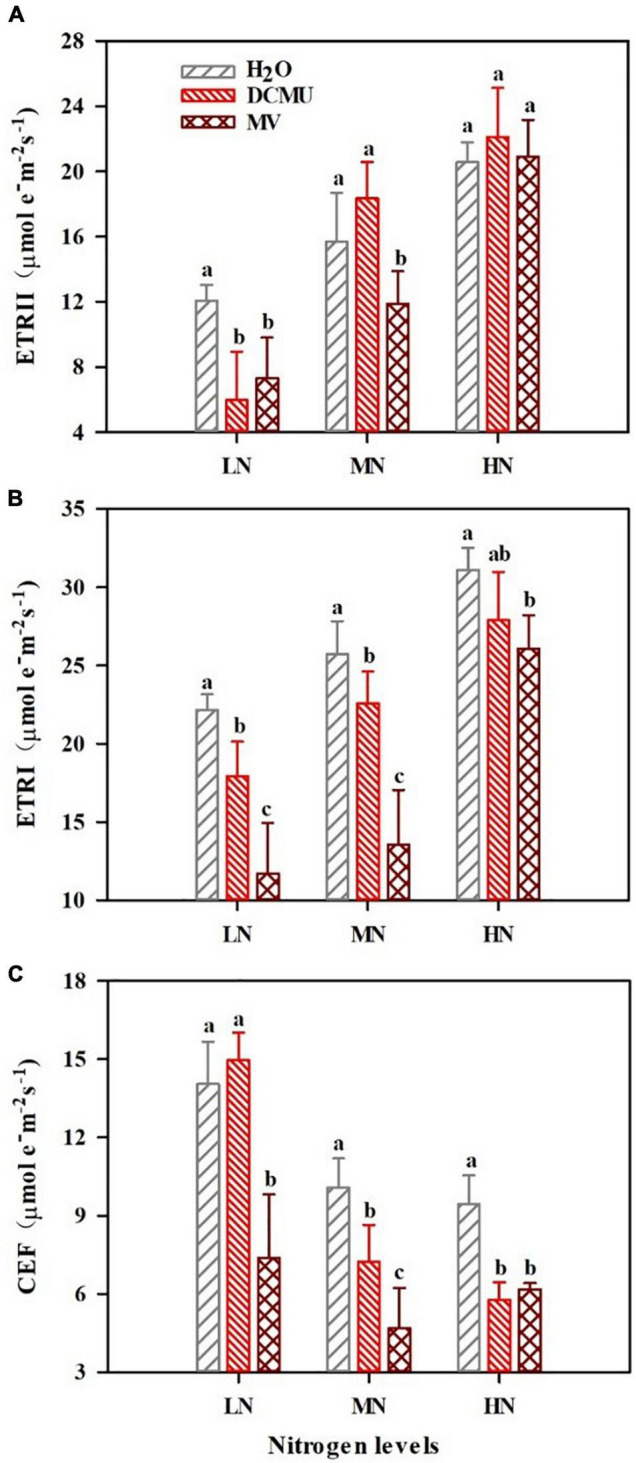
Effects of 3-(3,4-dichlorophenyl)-1,1-dimethyl urea (DCMU) and methyl viologen (MV) on ETRII, ETRI, and cyclic electron flow (CEF) in leaves of *Panax notoginseng* grown under different nitrogen regimes. **(A)** ETRII is the electron transport rate of PSII. **(B)** ETRI is the electron transport rate of PSI. **(C)** CEF is the cyclic electron flow. Values for each point were mean ± SD (*n* = 5). Different letters indicate significant differences among treatments (*p* < 0.05).

There were considerable differences in the photosynthetic electron transport under samples treated with DCMU and MV (Figure 4; *p* < 0.05). Electron transport rate of PSI (ETRI) and electron transport rate of PSII (ETRII) significantly reduced in LN-grown plants treated with DCMU (Figures 4A,B; *p* < 0.05). ETRI and CEF declined in MN-grown plants treated with DCMU (Figures 4B,C). In MN and HN plants treated with DCMU, the values of CEF decreased by 28.2 and 38.8%, respectively (Figure 4C). Meanwhile, MV-treated plants showed lower levels of ETRI and ETRII under N regimes (Figures 4A,B). In LN, MN, and HN plants treated with MV, the values of CEF reduced by 47.4, 53.6, and 34.6%, respectively (Figure 4C).

### Responses of the OJIP Kinetic Curve to 3-(3,4-Dichlorophenyl)-1,1-Dimethyl Urea and Methyl Viologen Under N Regimes

**FIGURE 5:**
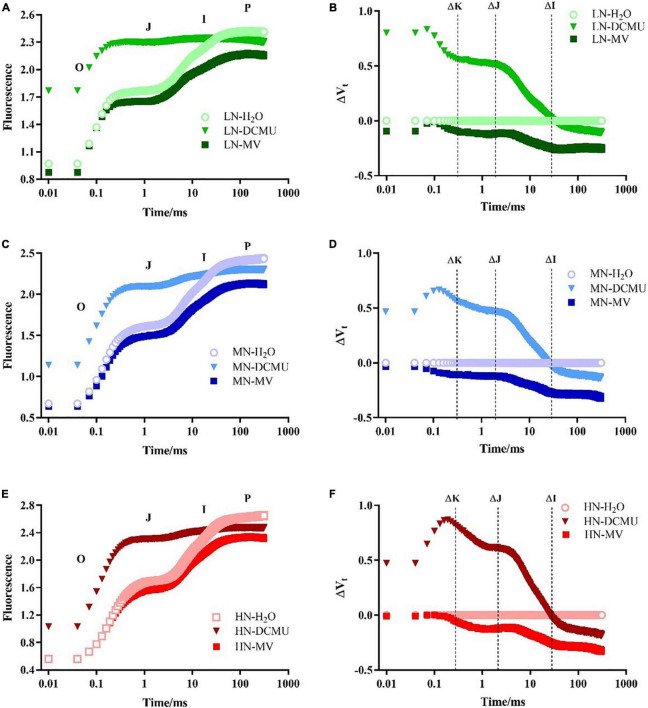
Effects of 3-(3,4-dichlorophenyl)-1,1-dimethyl urea (DCMU) and methyl viologen (MV) on chlorophyll fluorescence transients in leaves of *Panax notoginseng* grown under different nitrogen (N) regimes. After infiltration with chemical reagents (H_2_O, DCMU, and MV) in darkness for 12 h. OJIP kinetic curves were measured as described in the section “Materials and Methods.” Panels **(A,C,E)** represent the N levels of LN, MN, and HN, respectively. O, J, I, and P phases represent the fluorescence at T = 20 μs, 2, 30, and 300 ms, respectively. Effect of N levels LN **(B)**, MN **(D),** and HN **(F)** on relative variable fluorescence (ΔV_t_) of *Panax notoginseng* after infiltration with chemical reagents (H_2_O, DCMU, and MV) in darkness. ΔV_t_ = V(treatment) – V(control), V(treatment) is the fluorescence of *P. notoginseng* treated with DCMU or MV; V(control) is the fluorescence of *P. notoginseng* treated with H_2_O. ΔK, ΔJ, and ΔI represent the relative variable fluorescence at T = 300 μs, 2, and 30 ms, respectively. Values for each point were means (*n* = 5).

**FIGURE 6:**
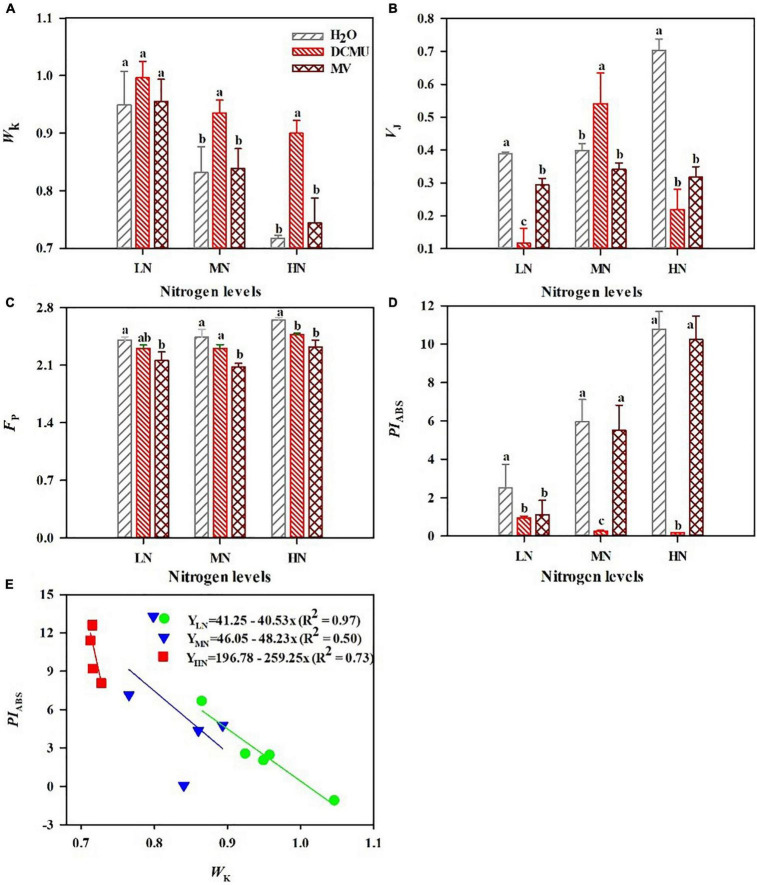
Effects of 3-(3,4-dichlorophenyl)-1,1-dimethyl urea (DCMU) and methyl viologen (MV) on *W*_K_ **(A)**, *V*_J_ **(B)**, *F*_P_ **(C)**, and *PI*_ABS_ **(D)** in leaves of *Panax notoginseng* grown under different nitrogen regimes. *W*_K_ is the K phase in OJIP kinetic curves **(A)**; *V*_J_ is the relative variable fluorescence intensity at the J-step **(B)**; *F*_P_ is the fluorescence intensity at the P-step **(C)**; *PI*_ABS_ is the performance index on absorption basis **(D)**. Values for each point were mean ± SD (*n* = 5). **(E)** Figures represent the correlation between *PI*_ABS_ and *W*_K_. Letters indicate significant differences at *p* < 0.05 according to Duncan’s multiple range tests.

After spraying the electron transport inhibitor, the OJIP curve changes significantly (Figure 5; *p* < 0.05). DCMU-treated samples showed higher K phase (300 μs) and J phase (2 ms) in the OJIP kinetic curve than those treated with H_2_O and MV (Figure 5). Spraying of MV has no significant effect on the OJIP kinetic curve, but the minimum value of I phase (30 ms) appeared in MV-treated samples (Figure 5). IP phase was decreased in MV-treated *P. notoginseng* compared with H_2_O-treated plants, and the minimum value of P phase (*F*_P_) appeared in MV-treated samples (Figures 5, 6C). Further analysis of the JIP-test parameters showed that the photosynthetic characteristics of PSII were significantly affected after spraying the electron transport inhibitor (Figure 6; *p* < 0.05). *PI*_ABS_ (performance index for energy conservation from photons absorbed by PSII antenna to the reduction of Q_B_) and *V*_J_ (relative variable fluorescence at the J-step) decreased in LN- and HN-grown plants treated with DCMU and MV (Figures 6B,D), but *W*_K_ (the ratio of the variable fluorescent *F*_K_ occupying the *F*_J_–*F*_O_ amplitude) significantly increased in LN-grown plants treated with DCMU (Figure 6A; *p* < 0.05). MN-grown plants treated with DCMU had significantly higher *V*_J_ and *W*_K_ (Figures 6A,B; *p* < 0.05). Meanwhile, *PI*_ABS_ significantly declined in MN-grown plants treated with DCMU (Figure 6D; *p* < 0.05).

According to the above result, the *PI*_ABS_ and *W*_K_ correlated well with the N levels. To further assess the N tolerance of *P. notoginseng*, a model was developed based on the parameter *PI*_ABS_ and *W*_K_ (Figure 6E). The *PI*_ABS_ values decreased linearly as the K-step level (*W*_K_) increased (Figure 6E). A significant negative linear correlation was observed between *PI*_ABS_ and *W*_K_ (Figure 6E). This linear relationship that the most important determinant of the PSII loss of function is the damage of OEC centers. The absolute value of the slope (*K*) of the relationship between *PI*_ABS_ and *W*_K_ quantifies plant sensitivity to N. The absolute value of the slope (*K*) is 40.53 (LN), 48.23 (MN), and 259.25 (HN), respectively. It is clear that a lower absolute value of slope has a stronger tolerance to nitrogen.

### Effects of 3-(3,4-Dichlorophenyl)-1,1-Dimethyl Urea and Methyl Viologen on the Distribution of Photosystem I Absorbed Light Energy in *Panax notoginseng* Leaves Under N Regimes

**FIGURE 7:**
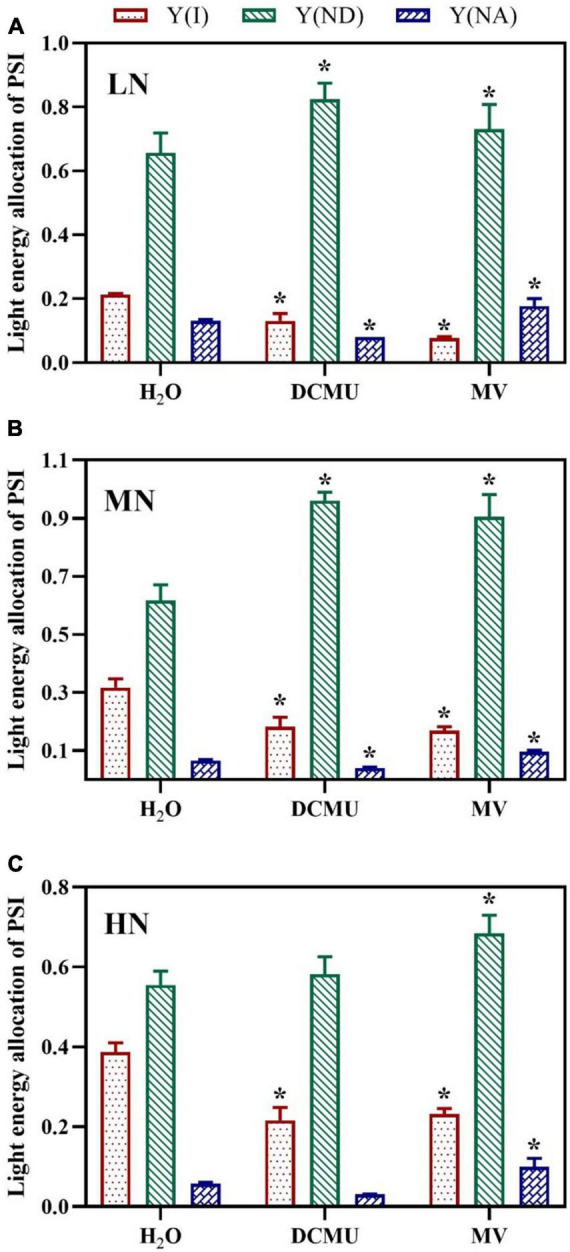
Effects of 3-(3,4-dichlorophenyl)-1,1-dimethyl urea (DCMU) and methyl viologen (MV) on light energy allocation of PSI in *Panax notoginseng* grown under different nitrogen regimes (light adapted condition). Panels **(A–C)** represent the nitrogen (N) levels of LN, MN, and HN, respectively. Y(I) is the quantum yield of PSI; Y(ND) is the donor side limitation of PSI; Y(NA) is the acceptor side limitation of PSI. Values for each point were means ± SD (*n* = 5). Significant differences are indicated by asterisks (ANOVA; *p* < 0.05). LN, low N; MN, moderate N; HN, high N; MV, methyl viologen.

Compared with the H_2_O-treated leaves, values for Y(I) (quantum yield of PSI) in plants of leaves treated with DCMU and MV were decreased (Figure 7). Y(I) was lowest when the plant was exposed to LN, and the minimum value of Y(I) appeared in MV-treated plants (Figure 7A). The samples treated with DCMU and MV had significantly higher Y(ND) (donor side limitation of PSI) than the H_2_O-treated samples (Figure 7; *p* < 0.05). Meanwhile, MN-grown plants showed the highest levels of Y(ND) (Figure 7B). The value of Y(NA) (acceptor side limitation of PSI) was kept at low levels in samples treated with H_2_O, DCMU, and MV, whereas the LN-grown plants showed higher Y(NA) (Figure 7). Concurrently, Y(NA) considerably enhanced in the MV-treated plants (Figure 7). Y(NA) was zero in the DCMU-treated plants (Figure 7).

### Response of the Redox Kinetics of P700 to 3-(3,4-Dichlorophenyl)-1,1-Dimethyl Urea and Methyl Viologen in N Regimes

**FIGURE 8:**
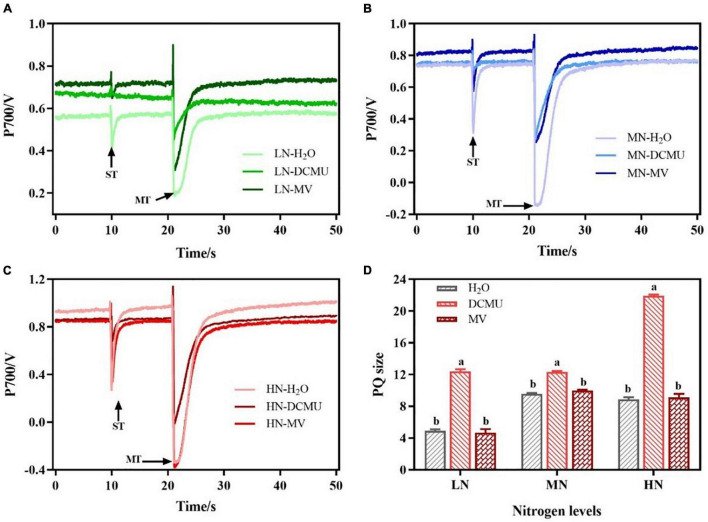
Effects of 3-(3,4-dichlorophenyl)-1,1-dimethyl urea (DCMU) and methyl viologen (MV) on plastoquinone (PQ) in leaves of *Panax notoginseng* grown under different nitrogen regimes. Panels **(A–C)** represent the nitrogen (N) levels of LN, MN, and HN, respectively. Panel **(D)** is the PQ pool size. Values for each point were mean ± SD (*n* = 5). Letters indicate significant differences at *p* < 0.05 according to Duncan’s multiple range tests. ST, single-turn saturation pulse; MT, multi-turn saturation pulse; LN, low N; MN, moderate N; HN, high N.

The P700 signal was determined during ST followed by MT in the presence of far-red background light and used ST areas/MT areas to characterize PQ pool size and the redox state (Savitch et al., 2001; Figure 8). In LN-, MN-, and HN-grown plants treated with DCMU, the PQ pool size increased by 60.3, 22.3, and 59.5%, respectively (Figure 8D). However, there were no significant differences in PQ pool size between MV-treated and H_2_O-treated plants (Figure 8D; *p* > 0.05).

### Effects of 3-(3,4-Dichlorophenyl)-1,1-Dimethyl Urea and Methyl Viologen on Leaf Antioxidant Enzyme Activity Under N Regimes

**FIGURE 9:**
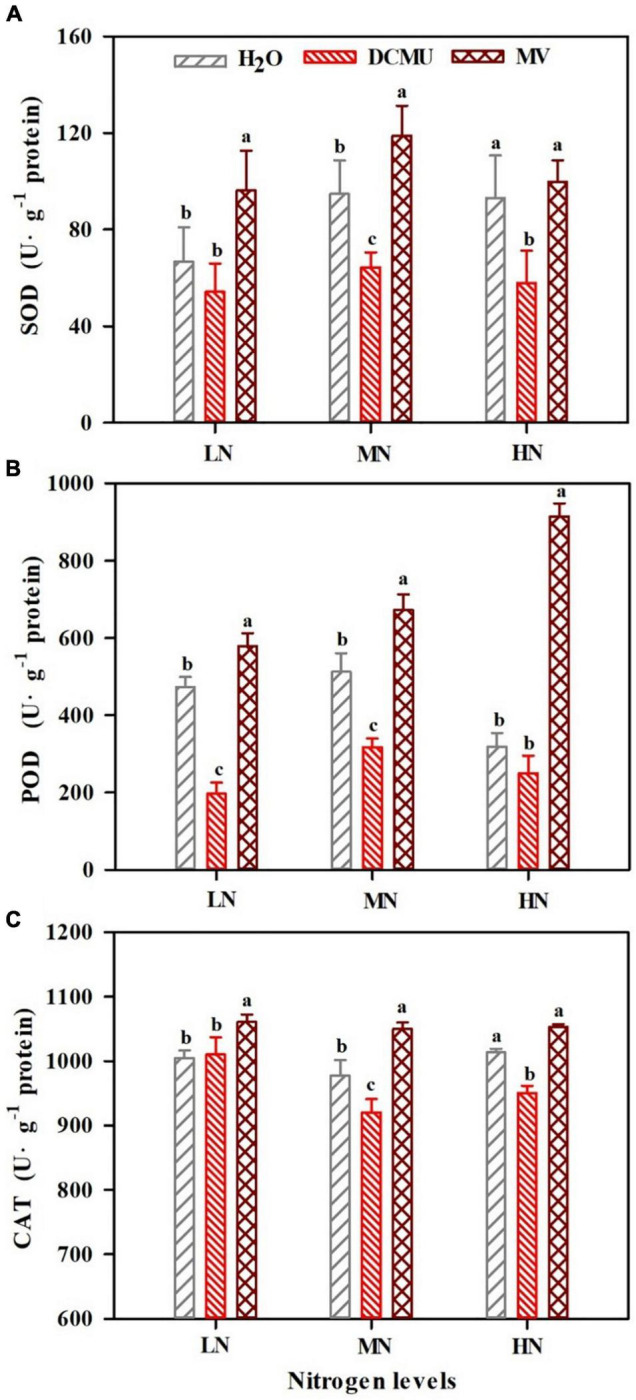
Effects of 3-(3,4-dichlorophenyl)-1,1-dimethyl urea (DCMU) and methyl viologen (MV) on superoxide dismutase (SOD) **(A)**, peroxidase (POD) **(B)**, and catalase (CAT) **(C)** in leaves of *Panax notoginseng* grown under different nitrogen regimes. Values for each point were mean ± SD (*n* = 5). Letters indicate significant differences at *p* ≤ 0.05 according to Duncan’s multiple range tests.

Activity of antioxidant enzymes in *P. notoginseng* showed significant differences between treatments (Figure 9; *p* < 0.05). SOD and CAT activity substantially increased in LN- and MN-grown plants treated with MV (Figures 9A,C; *p* < 0.05). Interestingly, SOD and CAT activity greatly reduced in MN- and HN-grown plants treated with DCMU (Figures 9A,C; *p* < 0.05). All these MV-treated samples showed high levels of POD activity, and the highest activity was found in HN-grown plants (Figure 9B). Meanwhile, POD activity markedly declined in LN- and MN-grown plants treated with DCMU (Figure 9B; *p* < 0.05).

## Discussion

In this study, we examined the roles of electron transport in PSI and PSII photoinhibition in the N-sensitive species *P. notoginseng* under N stress by treatments with diuron (DCMU) and MV. DCMU affects the primary photochemistry and blocks the electron transport from Q_A_ to the secondary quinone acceptor Q_B_ by interacting with D1 protein (Chen et al., 2007; Guo et al., 2020), whereas MV captures electrons from PSI ahead of FNR at almost the same rate as PSII is pumping them to PSI (Schansker et al., 2005). We found that PSI and PSII activities were considerably inhibited in LN plants as comparison with the other two treatments. Furthermore, LEF and CEF were an important determinant of PSI and PSII photoinhibition in *P. notoginseng* grown under LN. Our present study has revealed some insights into the mechanism of N stress-induced PSII and PSI photoinhibition in an N-sensitive species.

### Non-optimal N Regimes Inhibit Photosynthetic Capacity in an N-Sensitive Species

Nitrogen is one of the most important elements for plant photosynthesis as N is the main constituent of Rubisco, Chl, and photosynthetic proteins (Li et al., 2013; Mu and Chen, 2021). N uptake and utilization significantly affects photosynthetic efficiency (Evans and Clarke, 2019). Evidence shows that photosynthetic efficiency was increased with the increase of NUE in *Platycodon grandiflorus* and *Abrus cantoniensis* (Hu, 2006; Duan et al., 2015). However, root biomass and NUE increased in LN plants (Figure 1), whereas *P*_n_, *F*_v_/*F*_m_, and *P*_m_ declined (Figure 2). *P. notoginseng* promotes the accumulation of root biomass by increasing the NUE for survival under LN, but it does not contribute to photosynthesis. This has been confirmed by the performance observed in *A. thaliana* grown under N deficiency (Jia et al., 2020). Additionally, photosynthesis is closely related to leaf N and Chl contents (Rogers et al., 2017; Fraser, 2020). High SLN and Chl content induce the increase of light harvesting (Hikosaka, 2004). Our results are consistent with the previous studies that show SLN, Chl, *P*_n_, *F*_v_/*F*_m_, and *P*_m_ were reduced in *Isatis indigotica* and *P. notoginseng* under LN condition, thereby reducing light harvest and inhibiting photosynthesis (Guan et al., 2018; Figures 1, 2). Numerous studies have shown that the continued increase in SLN and Chl contents are detrimental to the balance of light harvesting and utilization, leading to a decrease in photosynthetic capacity (Li Y. et al., 2009; Moriwaki et al., 2019; Fraser, 2020). SLN, Chl, leaf, and stem biomass increased in HN plants, whereas *P*_n_ and NUE decreased (Figures 1, 2A), indicating photosynthetic efficiency was suppressed in HN condition. Meanwhile, the decrease in Rubisco and *P*_n_ has been observed in *P. notoginseng* grown under HN level (Figures 2A,D). The reduction in Rubisco activity and photosynthetic efficiency has been recorded in plants such as *Malus domestica* and *Pseudotsuga menziesii* var. *menziesii* (Cheng and Fuchigami, 2000; Manter et al., 2005). These results suggest that relatively less N operates on photosynthesis, when a high proportion of N served as N storage in HN-grown plants (Liu et al., 2018). Excessed SLN and Chl might cause the imbalance between the harvesting and utilization in light, thus leading to reduced photosynthetic capacity in the HN plants, as has been confirmed in soybean (*Glycine max*) by Jiang et al. (2006). Overall, plants absorb N mainly for the accumulation of root biomass in LN condition, whereas HN-grown plants absorb N mainly for the increase of aboveground biomass and N content. However, the non-optimal SLN, Chl, and Rubisco activity in the HN and LN plants caused an imbalance between light energy capture and utilization, thereby inhibiting photosynthetic efficiency.

### Higher Cyclic Electron Flow Cannot Prevent Photosystem I and Photosystem II From Photoinhibition Under Low N Condition

Photoinhibition of photosystems (PSI and PSII) occurs when the light energy absorbed by antenna pigment exceeds the capacity of photosynthetic apparatus (Sonoike, 2011; Tyystjärvi, 2013). LEF and CEF can rebalance ATP/NADPH and alleviate photoinhibition (Lu et al., 2020). *F*_v_/*F*_m_ and *P*_m_ were reduced by adding DCMU and MV that determine whether or not CEF and LEF are initiated (Figure 3). This has been confirmed by the performance observed in *Fragaria ananassa*, *Lycopersicon esculentum*, *Dalbergia odorifera*, *Erythrophleum guineense*, and *P. rubra* treated with DCMU and MV (Huang, 2012; Liu, 2015; Huang et al., 2016b; Lu, 2016). *F*_v_/*F*_m_ and *P*_m_ were significantly decreased in LN-grown plants treated with DCMU and MV (Figure 3). These results suggest that PSI and PSII are sensitive to LN, and MV and DCMU mainly inhibit the activities of PSI and PSII, respectively. On the other hand, it has been reported that *Eupatorium adenophorum* and *Cerasus cerasoides* adapt to the fluctuating light by enhancing electron transport to improve the utilization of light energy (Wang et al., 2004; Yang et al., 2019). ETRI, ETRII, and *F*_v_/*F*_m_ showed a decrease in LN-grown *P. notoginseng* (Figures 3A, 4A,B). We speculate that the reduction in PSII activity leads to the decrease in linear electron transport (ETRI and ETRII) when PSII photoinhibition occurs in LN-grown plants. The similar results have been recorded in *P. notoginseng* grown under low light and HN (Huang et al., 2018; Cun et al., 2021). In addition, CEF plays an important role in the adaptation of plants to environmental stress (Nawrocki et al., 2019; Sagun et al., 2019). The activation of CEF increases the adaptability of *Tradescantia fluminensis* leaves to high light stress (Kalmatskaya et al., 2020). CEF was higher in LN plants than in the HN and MN plants (Figure 4C). There were no significant differences in the CEF under LN-grown plants treated with H_2_O and DCMU, but CEF, *F*_v_/*F*_m_, and *P*_m_ were significantly reduced in LN-grown plants treated with MV (Figures 3, 4C). Therefore, higher CEF cannot prevent PSI and PSII from photoinhibition under LN condition.

### Lower Linear Electron Transport Induces Photodamage to Photosystem II Donor Side Under Low N Condition

The damage of PSII OEC and production of ROS are regarded as the two main causes of PSII photodamage (Li et al., 2018). The appearance of the K phase in OJIP is related to the damage of the PSII donor side, particularly the OEC (Strasser, 1997; Strasser et al., 2000, 2004; De Ronde et al., 2004; Li P. et al., 2009). The free radicals generated by excess excitation energy could damage the RC of PSII (excess-energy hypothesis; Krieger-Liszkay et al., 2008; Takahashi and Murata, 2008). *F*_v_/*F*_m_ was reduced in *P. notoginseng* grown under LN (Figure 3A), and there was no significance in *W*_K_ in LN-grown plants treated with MV and H_2_O (Figures 5, 6A). It indicates that additional free radicals cannot directly cause PSII photoinhibition in *P. notoginseng* under LN. Therefore, free radicals are not the main cause of photoinhibition of PSII under LN. Moreover, the photodamage of PSII occurs first at the OEC and then acts on the RC (Mn hypothesis; Murata et al., 2007; Nishiyama et al., 2011). *F*_v_/*F*_m_ and *PI*_ABS_ were decreased (Figures 3A, 6D), and *W*_K_ was increased in LN-grown plants treated with H_2_O, DCMU, and MV (Figure 6A). It might be speculated that LN-induced photoinhibition of PSII might be caused by the photodamage to the donor side of PSII OEC (Li P. et al., 2009). This has also been confirmed by the performance as has been observed in *Vitis vinifera* and *Hordeum vulgare* grown under drought stress (Christen et al., 2007; Oukarroum et al., 2007). A significant negative linear correlation was observed between *PI*_ABS_ and *W*_K_ (Figure 6E), this further confirms that the most important determinant of the PSII loss of function is the damage of OEC centers (Chen S. et al., 2016). Additionally, the most sensitive characteristic parameter *PI*_ABS_ and *W*_K_ of the OJIP kinetic curve is a useful and practical method for screening and assessing plant stress tolerance (Oukarroum et al., 2007; Boureima et al., 2012; Silvestre et al., 2014). This technique has successfully been applied to evaluate salinity sensitivity in *Vigna radiata* and *Brassica juncea* and heat tolerance in *Ageratina adenophora* (Misra et al., 2001; Chen S. et al., 2016). The absolute value of the slope (*K*) is 40.53 (LN), 48.23 (MN), and 259.25 (HN), respectively (Figure 6E). It is clear that *P. notoginseng* is tolerant to LN and sensitive to HN. This is consistent with our previous research results (Zhang J.Y. et al., 2020). Photodamage of PSII occurs when the rate of PSII photodamage is greater than the rate of PSII repairment (Miyata et al., 2015; Ueno et al., 2016; Takahashi et al., 2019). It has been reported that LEF facilitates the PSII repairment through a rapid formation of ΔpH (the proton gradient across the thylakoid membranes) in *A. thaliana* and *D. odorifera* grown under high-light stress (Huang, 2012; Yamada et al., 2020). ETRI and ETRII decreased in LN plants (Figures 4A,B). These results indicate that lower LEF causes the formation of ΔpH and ATP to be hindered, thereby inhibiting the repairment of PSII under LN. In other words, the inhibition of linear electron transport may cause damage to the OEC on the donor side of PSII under LN condition. *V*_J_ had the minimum value in LN plants treated with DCMU (Figures 5, 6B), and the DCMU-treated leaves still have the OJIP kinetics under MN and HN (Figures 5, 6B). The results imply that DCMU has inhibited all PSII RCs under LN condition, and the deactivation of PSII RC could produce a large number of free radicals (Domonkos et al., 2013; Tyystjärvi, 2013). However, the spraying of DCMU would weaken the inhibition of PSII RCs in *P. notoginseng* grown under HN and MN. The generation of ROS and the damage of OEC leads to PSII photodamage in *P. notoginseng* grown under LN. Overall, free radical production only inhibits PSII repairment and does not cause PSII damage, and lower LEF induces photodamage to PSII donor side under LN condition.

### Over-Reduction of Photosystem I Acceptor Side Under Low N Condition

A reaction between reduced iron–sulfur centers and hydroxyl peroxide generates hydroxyl radicals that cause oxidative damage to PSI complexes (Sonoike, 2011). The over-reduction of PSI acceptor side and production of hydroxide at PSI acceptor side are regarded as two main causes of PSI photoinhibition (Munekage et al., 2002; Tikkanen et al., 2014). Water–water cycle induces the production of hydroxyl peroxide at PSI acceptor side in *C. sativus*, *S. oleracea*, *A. thaliana*, and *S. lycopersicum* under chilling temperature or high light, which further leads to PSI photoinhibition (Sonoike, 1996; Zhang and Scheller, 2004; Lu et al., 2017). Y(NA) decreased with the increase of N levels, but Y(ND) was significantly higher than Y(NA), and MV-treated plants showed higher Y(NA) (Figure 7). These results indicate that the donor side limitation-induced superoxide anions are generated at the PSI receptor side under LN, which leads to excessive reduction of the PSI receptor side, thereby causing PSI photoinhibition. Moreover, it has been observed that the excitation of CEF might maintain the low reduction state of the PSI receptor side and the high oxidation state of P700 to prevent PSI from damage in *P. vulgaris*, *Triticum aestivum*, and *C. sinensis* grown under N deficient (Antal et al., 2010; Lin et al., 2016; Li H. et al., 2021). The size of PQ pool in LN plants was reduced, and the CEF was increased, and there were no significant differences in PQ pool size between MV-treated and H_2_O-treated plants (Figures 4C, 8D). Therefore, the higher CEF-induced reduction in PQ pool not only reduces the acidification of the lumen, thereby preventing photodamage to PSI, but also slows the rate of damage to PSII OEC under LN, as has been confirmed in *P. vulgaris* and *C sinensis* grown under N starvation (Antal et al., 2010; Lin et al., 2016). Nevertheless, higher PQ pool size was recorded in DCMU-treated plants. We speculate that lower CEF cannot alleviate the over-reduction of the PQ pool in DCMU-treated plants and thus inhibit the photosynthetic capacity (Figure 8D), as has been confirmed by Allen (2003). On the other hand, antioxidant enzymes (SOD, POD, CAT, etc.) play an important role in scavenging ROS (Foyer et al., 1994). It has been reported that MV significantly increases the activity of CAT and POD in *Salvia miltiorrhiza*, whereas DCMU inhibits the effects of MV (Xing et al., 2014). These are in line with results obtained in the present study that MV significantly enhanced the activity of SOD, CAT, and POD in *P. notoginseng*, whereas DCMU suppressed the activity of antioxidant enzyme (Figure 9). Enhanced antioxidant activity protects PSI from photodamage in *P. notoginseng* grown under LN. Therefore, over-reduction of PSI acceptor side is the main cause of PSI photoinhibition under LN condition, and higher CEF and activity of antioxidant enzyme protect PSI from photodamage.

## Conclusion

**FIGURE 10:**
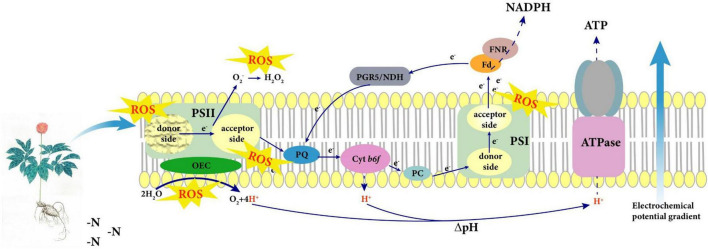
A model was proposed for the adaptation strategy of photosystem in the nitrogen (N)-sensitive plants represented by *Panax notoginseng* under low N (LN) condition. The lower linear electron flow (LEF) leads to the hindrance of the formation of ΔpH and ATP under LN, thereby damaging the donor side of the PSII oxygen-evolving complex (OEC). However, the activation of cyclic electron flow (CEF) slows down the damage rate of PSII under LN. Additionally, the over-reduction of PSI acceptor side is the main cause of PSI photoinhibition in N-sensitive species *P. notoginseng* under LN, and higher CEF and antioxidant enzyme activity protects PSI from photodamage. Craquelure represents the damage of photosystem.

Non-optimal N regimes significantly inhibit photosynthetic capacity of the N-sensitive species such as *P. notoginseng*. Plants absorb N mainly for the accumulation of root biomass in LN condition. A high SLN and Chl might cause the imbalance between light capture and utilization, thus reducing photosynthetic carboxylation capacity in the HN plants. A model was proposed for the adaptation strategy of photosystem in the N-sensitive plants represented by *P. notoginseng* under LN condition (Figure 10). The lower LEF leads to the hindrance of the formation of ΔpH and ATP in LN plants, thereby damaging the donor side of the PSII OEC. The over-reduction of PSI acceptor side is the main cause of PSI photoinhibition under LN condition. Additionally, higher CEF and activity of antioxidant enzyme not only protected PSI from photodamage but also slowed down the damage rate of PSII in *P. notoginseng* grown under LN.

## Data Availability Statement

The original contributions presented in the study are included in the article/Supplementary Material, further inquiries can be directed to the corresponding author.

## Author Contributions

J-WC directed the whole process of the experiment and also made suggestions for the writing of the manuscript. ZC participated in the whole experiment, analyzed the relevant experimental data, and wrote the manuscript. H-MW, J-YZ, and S-PS measured the light absorption in photosystem I and chlorophyll fluorescence. H-MW and JH participated in the determination of photosynthetic pigment content and steady-state gas exchange measurements. All authors contributed to the article and approved the submitted version.

## Conflict of Interest

The authors declare that the research was conducted in the absence of any commercial or financial relationships that could be construed as a potential conflict of interest.

## Publisher’s Note

All claims expressed in this article are solely those of the authors and do not necessarily represent those of their affiliated organizations, or those of the publisher, the editors and the reviewers. Any product that may be evaluated in this article, or claim that may be made by its manufacturer, is not guaranteed or endorsed by the publisher.
