# Supplementary material for: Responses of Linear and Cyclic Electron Flow to Nitrogen Stress in an N-Sensitive Species Panax notoginseng
Source: Front Plant Sci. 2022 Feb 15;13:796931. doi: 10.3389/fpls.2022.796931 (PMC8885595; doi:10.3389/fpls.2022.796931)

# SUPPLEMENTARY FIGURE

**FIGURE S1** Effects of MV **(A-B)** and DCMU **(C-D)** on *F*_v_/*F*_m_ and *P*_m_ in leaves of *Panax notoginseng*. The decrease of *F*_v_/*F*_m_ and *P*_m_ is a characteristic of photoinhibition in PSII and PSI, respectively. The electron transport inhibitor concentration with the greatest inhibition of PSI and PSII activity was selected for treatment of *P. notoginseng* leaves. Values for each point were means ± SD (*n* = 5). Letters indicate significant differences at *P* < 0.05 according to Duncan’s multiple range tests.

# Figures S1


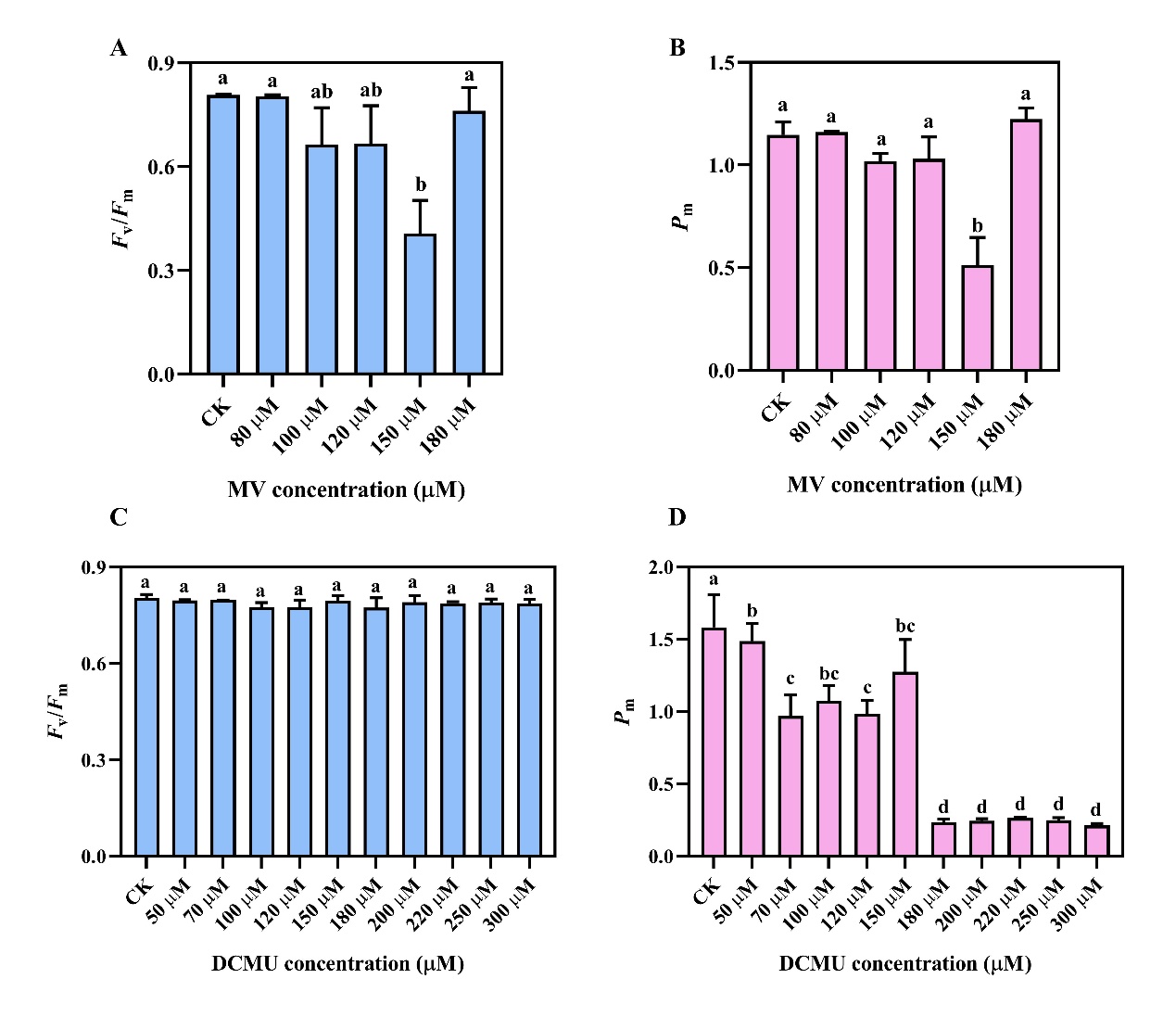

Supplement: Supplementary file 1 [file Data_Sheet_1.docx]
