# Supplementary material for: Responses of Linear and Cyclic Electron Flow to Nitrogen Stress in an N-Sensitive Species Panax notoginseng
Source: Front Plant Sci. 2022 Feb 15;13:796931. doi: 10.3389/fpls.2022.796931 (PMC8885595; doi:10.3389/fpls.2022.796931)
Supplement: Supplementary file 2 [file Data_Sheet_2.docx]

# SUPPLEMENTARY TABLE

**TABLE S1 Formulae used to calculate chlorophyll fluorescence parameters**

| Parameter | Description |
| --- | --- |
| *F*_v_/*F*_m_=(*F*_m_-*F*_o_)/*F*_m_ | The maximum photochemistry efficiency of PSII |
| Y(II)=(*F*_m_`-*F*_s_)/*F*_m_` | The effective photochemical quantum yield of PSII |
| Y(NA)=(*P*_m_-*P*_m_`)/*P*_m_ | The acceptor side limitation of PSI |
| Y(ND)=1-P700red | The donor side limitation of PSI |
| Y(I)=1−Y(ND)−Y(NA) | The effective photochemical quantum yield of PSI |
| ETRI=Y(I)×PPFD×0.84×0.5 | The electron transport rate of PSI |
| ETRI=Y(II)×PPFD×0.84×0.5 | The electron transport rate of PSII |
| CEF=ETRI-ETRII | Cyclic electron transport rate (Huang et al., 2012b) |
| *F*_t_ | Fluorescence at time *t* after onset of actinic illumination |
| *F*_O_≌*F*_20 μs_ | Minimal fluorescence, when all RCs are open |
| *F*_K_≡*F*_300 μs_ | Fluorescence intensity at the K-step (300 μs) of OJIP |
| *F*_J_≡*F*_2 ms_ | Fluorescence intensity at the J-step (2 ms) of OJIP |
| *F*_I_≡*F*_30 ms_ | Fluorescence intensity at the I-step (30 ms) of OJIP |
| *F*_P_≡*F*_300 ms_= *F*_M_ | Maximal recorded fluorescence intensity, at the peak P of OJIP |
| *V*_t_= (*F*_t_- *F*_O_)/ (*F*_M_- *F*_O_) | Relative variable fluorescence at time *t* |
| *V*_J_= (*F*_J_- *F*_O_)/ (*F*_M_- *F*_O_) | Relative variable fluorescence at the J-step |
| *W*_K_= (*F*_K_- *F*_O_)/ (*F*_J_- *F*_O_) | Relative variable fluorescence at the K-step to the amplitude *F*_J_- *F*_O_ |
| *PI*_ABS_=10×*F*_M_×(1- *V*_J_)/( *F*_O_×*V*_J_×2×*M*_o_) | Performance index (potential) for energy conservation from photos absorbed by PSII to the reduction of intersystem electron acceptors |
